# The Mediating Roles of Resilience and Flow in Linking Basic Psychological Needs to Tertiary EFL Learners’ Engagement in the Informal Digital Learning of English: A Mixed-Methods Study

**DOI:** 10.3390/bs15010085

**Published:** 2025-01-18

**Authors:** Yang Gao, Xiaochen Wang, Barry Lee Reynolds

**Affiliations:** 1 School of Foreign Studies, Xi’an Jiaotong University, Xi’an 710049, China; gaoyang666@xjtu.edu.cn; 2 Faculty of Education, University of Macau, Macau SAR, China; 3 Centre for Cognitive and Brain Sciences, University of Macau, Macau SAR, China

**Keywords:** informal digital learning of English, resilience, flow, engagement, basic psychology needs

## Abstract

Resilience and flow are crucial in language education, yet most research focuses on formal learning environments, with limited studies on their impact in informal settings. This study explores the relationship between basic psychological needs and engagement in the context of informal digital English learning (IDLE). Using a mixed-methods design, data were collected from 512 Chinese EFL learners. Structural equation modeling and NVivo analysis were applied to quantitative and qualitative data, respectively. The findings reveal that resilience fully mediates the relationship between basic psychological needs and engagement, serving as an adaptability enhancer, persistence promoter, stress buffer, self-efficacy builder, and emotional regulation facilitator. Conversely, flow partially mediates this relationship, though perceived competence does not significantly predict flow in this context. Building on this, flow contributes as an intrinsic motivation driver, positive cycle creator, external pressure mitigator, and efficiency enhancer. This research underscores the important role of resilience and flow in IDLE among Chinese university EFL students. By highlighting these mediating roles, the study provides valuable insights for enhancing the effectiveness of IDLE experiences, contributing to the broader discourse on language education in the digital age.

## 1. Introduction

In the digital age, the landscape of English language learning is undergoing transformative shifts, and a vast number of university students are leveraging informal digital platforms to enhance their English proficiency outside formal educational settings (Lee, 2020; Rezai et al., 2024a, 2024b; Soyoof et al., 2023; Zhang & Liu, 2022). This evolving trend highlights the critical need to explore how these informal digital environments contribute to language acquisition, aligning with the preferences of digital natives who seek interactive, flexible, and readily accessible learning experiences (Liu & Wang, 2024; Rezai et al., 2024c). In this sense, researching Informal Digital Learning of English (IDLE) is important for bridging the gap between formal and informal learning, offering insights into integrated educational strategies that cater to a broader spectrum of learner needs and preferences (Soyoof et al., 2024). This approach not only promises to enhance engagement and improve language learning outcomes but also facilitates a seamless educational journey, blending structured classroom environments with the autonomy of self-directed digital learning (Lee & Dressman, 2018). Additionally, in the context of globalization, IDLE research is crucial for uncovering effective methods to improve English proficiency, serving as a gateway to cross-cultural communication and international opportunities for learners in non-English speaking regions (Lee, 2020).

In recent years, there has been a growing recognition of the pivotal role that affective factors play in language learning journals (Gao et al., 2024; Wang et al., 2024b, 2024a). Scholars have investigated various psychological aspects influencing language acquisition, including self-efficacy (Wang et al., 2023), willingness to communicate (Guo et al., 2023), well-being (Pan et al., 2023), motivation (Wang et al., 2022), and academic engagement (Wang & Kruk, 2024). Despite the importance of these factors, the roles of flow and resilience remain significant yet relatively underexplored in language learning. Most studies have primarily explored these two factors from the perspective of language teachers (Dai & Wang, 2023; Derakhshan et al., 2022), overlooking the effect of them on students in language learning contexts, especially in the informal settings.

To address the research gap, this study adopts a mixed-method approach to investigate the mediating roles of resilience and flow in the interplay between basic psychological needs and engagement within the context of IDLE. Specifically, it dissects these two variables into three sub-dimensions for in-depth analysis: basic psychological needs are categorized into autonomy, competence, and relatedness, while engagement is explored through its behavioral, emotional, and cognitive dimensions. The platforms discussed in this study primarily refer to Chinese social media platforms such as Rednote (Xiaohongshu), Weibo, and Tiktok (Douyin), which are commonly used by students for the informal digital learning of English. This research makes two distinctive contributions. First, it extends prior studies by exploring the relationships among basic psychological needs, flow, resilience, and engagement, specifically within informal digital learning settings, an area that remains underexplored. This focus broadens the understanding of psychological dynamics in IDLE, offering a nuanced perspective on how these factors interact. Second, it uniquely examines the mediating roles of flow and resilience, providing new insights into the mechanisms through which basic psychological needs influence learner engagement in digital environments.

## 2. Literature Review

### 2.1. IDLE

IDLE is defined as “self-directed English activities in informal digital settings, motivated by personal interests and undertaken independently” (Lee & Lee, 2021, p. 359). Building upon this definition, Zhang & Liu (2022) highlight that IDLE should be autonomous and occur naturally outside formal classroom settings, without structured guidance. Unlike the concept of digital wilds, which views online and out-of-class language or literacy learning primarily as social practices (Liu, 2022), IDLE encompasses a broader range of activities (Liu et al., 2024a, 2024b; Soyoof et al., 2024). It includes both form-focused tasks, such as studying vocabulary through mobile apps, and meaning-focused engagements, like participating in YouTube video comments (Lee, 2020; Liu et al., 2023b, 2024d). By leveraging the flexibility and extensive resources of digital environments, IDLE facilitates highly personalized and effective language learning (Liu et al., 2024c; Zhang & Liu, 2023). This approach not only broadens opportunities for language acquisition but also complements traditional methods, making it a versatile and impactful tool for promoting language development (Lee, 2020; Li et al., 2021).

Scholars have delved into IDLE from various perspectives to unravel its complexities and the broad impact it has on language learning. Research has assessed the efficacy of IDLE in enhancing language skills, highlighting its significant impact on learning outcomes (Lee, 2019; Sundqvist & Wikström, 2015). Another angle of investigation is how IDLE affects learner motivation and engagement with the English language, with studies indicating that digital platforms significantly influence learners’ enthusiasm and commitment (Almohesh & Altamimi, 2024; Wu, 2023). Furthermore, the role of IDLE in fostering cultural and intercultural competence has been explored, showing that exposure to diverse cultural content and interaction with native speakers and learners from various backgrounds through digital platforms enhances learners’ cultural understanding (Lee, 2020; Liu et al., 2023a). Additionally, the psychological aspects of IDLE, such as enjoyment, boredom, and language learning anxiety, have been examined to understand their influence on learners’ persistence in language learning via digital platforms (Lee et al., 2022; Taherian et al., 2023). Collectively, these studies contribute to a nuanced understanding of IDLE’s multifaceted role in language education, underscoring its potential to significantly improve language proficiency, motivation, cultural competence, and psychological well-being among learners.

### 2.2. Basic Psychological Needs: Autonomy, Relatedness, and Competence

The concept of basic psychological needs originates from Self-Determination Theory (SDT), a framework developed by Ryan & Deci (2000) to understand human motivation and well-being. Within SDT, three basic psychological needs are identified as essential for fostering personal growth, intrinsic motivation, and psychological health: autonomy, relatedness, and competence (Ryan & Deci, 2017, 2020). To be exact, autonomy is related to the need to be engaged with tasks in an autonomous manner within a context that is relevant to them. Relatedness is related to the need to be engaged in tasks that allow collaboration and communication with other students. Competence is related to the need of students to feel capable of successfully engaging in the learning process. Basic psychological needs have been widely applied to augment student learning in traditional classroom environments (Ryan & Deci, 2017, 2020). However, their application in informal settings remains relatively underexplored. Addressing this gap, Ryan & Deci (2020) have highlighted the importance of extending SDT research to examine how technological advancements, including e-learning platforms and remote learning tools, can facilitate the fulfillment of the three basic psychological needs, thereby enhancing student engagement.

### 2.3. Flow, or Resilience

Flow is the psychological condition of losing track of time and external factors due to total immersion in an activity (Csikszentmihalyi, 1997). Prior research has underscored the significant impact of basic psychological needs on flow. For instance, Stormoen et al. (2016) confirmed the correlation between basic psychological needs and the experience of flow within the context of high school physical education. Valenzuela et al. (2018) highlighted the pivotal roles that perceived autonomy and competence assume in the practice of conservatoire music. More recently, He et al. (2023) identified competence, autonomy, relatedness, and presence as key factors to facilitate the entry into a state of flow among college students in entrepreneurship courses. Against this backdrop, it is posited that in the realm of language learning, basic psychological needs similarly exert a substantial influence on the flow experience.

Resilience is defined as the sum of an individual’s abilities that allow him or her to bounce back from adversity and even thrive in the face of difficult times (Kim & Kim, 2017; Kim et al., 2018, 2019). In language learning, each factor of the basic psychological needs distinctly contributes to fostering resilience among learners. Specifically, competence fortifies learners against the discouragement that can accompany the language acquisition process. Moreover, autonomy supports resilience by fostering a sense of personal agency. Finally, relatedness offers emotional support that is crucial for resilience. These three needs, when adequately satisfied, can thus enhance a learner’s resilience, providing them with the psychological resources necessary to persist and thrive in the face of challenges (Liu & Huang, 2021; Neufeld & Malin, 2019).

Based on the above understanding, we propose the following hypotheses to further investigate the impact of basic psychological needs on flow and resilience:

**H1:**  *Perceived competence significantly predicts flow.*

**H2:**  *Perceived competence significantly predicts resilience.*

**H3:**  *Perceived autonomy significantly predicts flow.*

**H4:**  *Perceived autonomy significantly predicts resilience.*

**H5:**  *Perceived relatedness significantly predicts flow.*

**H6:**  *Perceived relatedness significantly predicts resilience.*

### 2.4. Engagement, Flow, and Resilience

In an educational context, student engagement is defined as the dedication of time and effort towards educational pursuits, or the act of actively participating in classroom activities (Kuh, 2009). Expanding on this definition, Hiver et al. (2021) proposed that engagement encompasses learners’ physical and mental investment in successfully completing a language learning task. This concept is recognized as multifaceted, encompassing behavioral, emotional, and cognitive dimensions of engagement (Dai & Wang, 2024; Guo & Wang, 2024; Lei et al., 2018; Wang et al., 2024c; Wang & Xue, 2024). Behavioral engagement in IDLE reflects learners’ involvement in digital English learning through apps, forums, and exercises, highlighting their self-motivated efforts to enhance language skills. Meanwhile, emotional engagement as operationalized in this study captures learners’ emotional responses, such as motivation and satisfaction, during their online English learning journey. Furthermore, cognitive engagement was operationalized as involving the use of critical thinking and self-regulated learning strategies to improve English proficiency in digital settings. Together, these dimensions illustrate a holistic view of engagement in the context of IDLE, emphasizing the active, emotional, and intellectual commitment of learners as they navigate and utilize digital environments to acquire language skills (Bond et al., 2020).

As previously mentioned, flow relates to the psychological condition of losing track of time and external factors due to total immersion in an activity (Csikszentmihalyi, 1997). This psychological state is characterized by a balance between the challenge of the task and the learner’s skill level, leading to optimal learning experiences (Ibrahim, 2020). When language learners achieve flow, they are more likely to engage deeply with the learning material, contributing to improved retention, higher motivation, and increased language proficiency (Liu & Song, 2021; Santosa, 2015). The correlation between flow and engagement underscores the importance of designing language learning activities that are both challenging and attainable, fostering a conducive environment for learners to experience flow (Özhan & Kocadere, 2020). This, in turn, can significantly impact their overall learning effectiveness and engagement, improving the engagement in the language learning journey (Hamari et al., 2016).

As discussed earlier, resilience embodies an individual’s abilities that allow him or her to bounce back from adversity and even thrive in the face of difficult times (Chu & Liu, 2022; Kim & Kim, 2017; Liu & Chu, 2022; Liu & Han, 2022). In the context of language learning, resilience emerges as a critical factor influencing learner engagement (Li, 2022; Shen, 2022; Yu et al., 2022). Research indicates that resilient learners display higher levels of engagement, as they are more likely to persist in their studies, employ adaptive coping strategies, and sustain motivation over time (Liu et al., 2022). This heightened engagement, fueled by resilience, not only enhances learners’ ability to overcome linguistic obstacles but also contributes to a deeper, more meaningful learning experience (Cena et al., 2023; Liu et al., 2023c). The interplay between resilience and engagement underscores the necessity of fostering a supportive learning environment that cultivates resilience, thereby enhancing learners’ engagement and overall language learning success (Ritonga et al., 2023). Based on the above understanding, we propose the following hypotheses to further investigate the impact of flow and resilience on language learning engagement:

**H7:**  *Flow significantly predicts behavioral engagement.*

**H8:**  *Flow significantly predicts cognitive engagement.*

**H9:**  *Flow significantly predicts emotional engagement.*

**H10:**  *Resilience significantly predicts behavioral engagement.*

**H11:**  *Resilience significantly predicts cognitive engagement.*

**H12:**  *Resilience significantly predicts emotional engagement.*

### 2.5. The Hypothesized Structural Model

**Figure 1:**
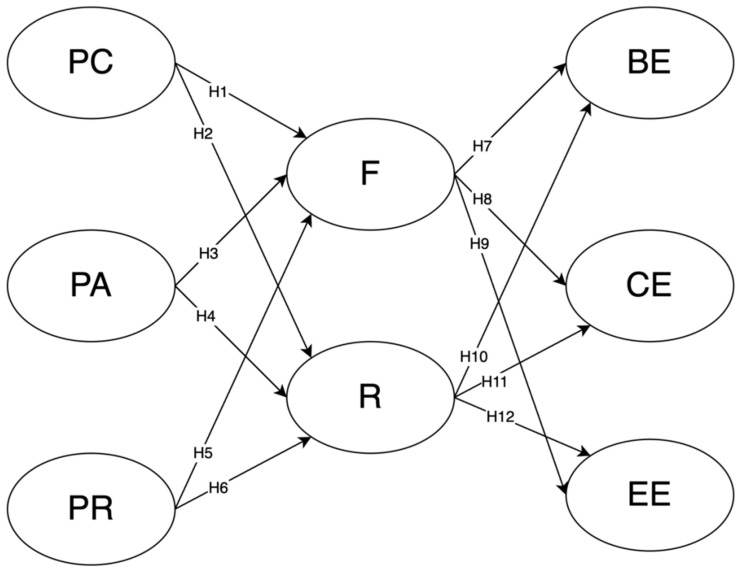
The hypothesized structural model. Note: PC = perceived competence; PA = perceived autonomy; PR = perceived relatedness; F = flow; R = resilience; BE = behavioral engagement; CE = cognitive engagement; EE = emotional engagement.

Figure 1 illustrates the hypothesized structural model, which examines the relationships among basic psychological needs (perceived competence, perceived autonomy, and perceived relatedness), mediators (flow and resilience), and engagement dimensions (behavioral, cognitive, and emotional engagement). Hypotheses H1 to H6 represent the predictive effects of basic psychological needs on flow and resilience, demonstrating how these psychological constructs support learners’ ability to engage deeply in learning activities. Hypotheses H7 to H12 test the influence of flow and resilience on different aspects of engagement, showcasing their roles in fostering active, cognitive, and emotional involvement in the informal digital learning of English (IDLE). This model provides a comprehensive framework to explore the mediating roles of flow and resilience in linking basic psychological needs to learner engagement.

### 2.6. Research Questions

Drawing from the previous literature, this study aims to investigate the following questions:

RQ1: Does resilience mediate the relationship between basic psychological needs and student engagement in IDLE, and what specific mediating role does it play?

RQ2: Does flow mediate the relationship between basic psychological needs and student engagement in IDLE, and what specific mediating role does it play?

## 3. Methodology

### 3.1. Research Design: An Explanatory Sequential Mixed-Method Design

This study employs a mixed-methods approach to explore the impact of resilience and flow on Chinese university English as a Foreign Language (EFL) students’ engagement in IDLE. Specifically, an explanatory sequential mixed-method design was used (Creswell et al., 2003). This design prioritizes quantitative data collection and analysis first. The initial quantitative phase (SEM) provides key findings and insights. The subsequent qualitative phase (NVivo) is then used to explain or interpret the quantitative results. In other words, SEM first quantitatively assesses the mediating role of resilience and flow between students’ basic psychological needs and their engagement levels. Based on the SEM results, qualitative interviews in Chinese are conducted to gain in-depth insights into significant relationships, such as how resilience and flow contribute to engagement in IDLE. Non-significant relationships were not explored further in the qualitative phase to maintain focus. This sequential explanatory design integrates quantitative findings with qualitative narratives to provide a comprehensive understanding of the mediating roles of resilience and flow in IDLE.

### 3.2. Participants

The participants comprised a diverse group of 512 Chinese university EFL students, with 60.5% females and 39.5% males. The age distribution was broad, with 23.8% being 19 or below, 41.2% between the ages of 20 and 22, and the remaining 35% aged 23 or above. Participants were drawn from various academic tiers, including 26.2% from Tier A (985 universities, representing top-tier institutions under the Chinese government’s ‘Project 985’ initiative), 32.4% from Tier B (211 universities, part of the ‘Project 211’ initiative to enhance education and research standards), and 41.4% from Tier C (all other universities not included in these initiatives). The sample spanned a range of academic disciplines, with the majority from the arts and humanities (43.8%), followed by the social sciences (21.1%), natural sciences (18.8%), engineering (13.3%), and a small fraction from other fields (3.0%). This composition ensures a wide perspective on the engagement in IDLE across different demographics and academic environments.

### 3.3. Instruments

#### 3.3.1. Basic Psychological Needs

The measurement of basic psychological needs utilized a modified version of the questionnaire developed by Ryan et al. (2006), which was adapted for a similar context by Jeon (2022). The questionnaire assessing basic psychological needs consisted of 9 items, equally divided into three dimensions: perceived autonomy, perceived competence, and perceived relatedness. Each dimension included 3 items, and responses were recorded using a 5-point Likert scale ranging from 1 to 5. For instance, a sample item for perceived autonomy was: “I experience a lot of freedom when learning English through social media”.

#### 3.3.2. Flow

The questionnaire employed in this research is adapted from the comprehensive framework developed by Hong et al. (2017), specifically tailored to explore the nuances of flow in informal digital English learning experiences. The flow questionnaire comprised 5 items aimed at evaluating participants’ immersion during informal digital English learning activities. The items were rated on a 5-point Likert scale from 1 to 5. A representative item was: “While using social media to learn English, I forgot about everything else”.

#### 3.3.3. Resilience

The questionnaire utilized in this research, adapted from the work of Smith et al. (2008), comprises a selected set of six questions specifically designed to measure resilience in the context of informal digital English learning experiences. Responses were assessed using a 5-point Likert scale from 1 to 5. An example of an item was: “I tend to bounce back quickly after hard times when using social media to learn English”.

#### 3.3.4. Engagement

In terms of engagement, we employed a questionnaire adapted from Deng et al. (2020) to measure engagement. The engagement questionnaire contained 9 items, evenly distributed across three dimensions: behavioral engagement, cognitive engagement, and emotional engagement (3 items per dimension). The items were rated on a 5-point Likert scale ranging from 1 to 5. A sample item was: “I try hard to do well when I learn English through social media compared to other platforms”.

#### 3.3.5. Semi-Structured Interviews

The semi-structured interviews aimed to explore the nuances of how resilience and flow contribute to engagement in IDLE, seeking to capture the depth and complexity of these phenomena among Chinese university EFL students. We primarily examine the following two questions: 

For resilience: Could you share the experience when you encountered a significant challenge or setback while learning English through digital platforms, and how you managed to maintain your learning process despite this obstacle?

For flow: Could you describe an instance where you were so absorbed in an online English learning activity that you lost track of time? What were you doing, and what do you think contributed to this immersive experience?

### 3.4. Data Collection

Data collection was carried out in two sequential phases to ensure a comprehensive integration of both quantitative and qualitative insights. The first phase involved the distribution of an online survey through the Wenjuanxin platform, chosen for its ability to reach a wide and diverse pool of potential participants. This approach facilitated extensive accessibility and encouraged broad participation. To qualify for the study, participants were required to meet specific criteria: they needed to demonstrate a clear understanding of the research objectives and voluntarily consent to participate. Additionally, only individuals with prior experience using digital tools for informal language learning were included, ensuring that the data collected was both relevant and meaningful to the study’s focus. Purposive sampling was employed to ensure that participants aligned with the study’s objectives and provided diverse perspectives relevant to IDLE. This targeted strategy enhanced the validity and applicability of the findings by capturing informed perspectives directly aligned with the research objectives.

In the second phase, a purposive sample of 20 respondents was selected from the survey participants for semi-structured interviews. Participants were purposively sampled based on specific criteria, including diversity in demographic variables (such as gender, academic background, and age group) and variability in survey responses regarding their engagement and experiences in the informal digital learning of English. This qualitative phase aimed to explore individual experiences in greater depth, providing insights that complemented the quantitative data. The interviews were conducted face-to-face in a private and quiet setting on university campuses, ensuring a comfortable and distraction-free environment. The first author conducted all interviews personally, adhering to a standardized protocol to maintain consistency and reliability across all sessions. Each session lasted approximately 30 min. There was a three-week interval between the completion of the survey phase and the start of the interviews. This interval allowed for sufficient time to analyze survey responses and purposively select participants who could provide rich qualitative insights. This approach ensured a richer and more contextual understanding of the participants’ engagement in IDLE.

### 3.5. Data Analysis

The data analysis for this study was executed using both quantitative and qualitative approaches, ensuring a comprehensive understanding of Chinese university EFL students’ engagement in IDLE. Quantitative data analysis was performed with SPSS 26.0 and AMOS 26.0. The initial stage involved data preparation, including the elimination of invalid responses and the identification of outliers through Mahalanobis distance. Skewness and kurtosis values were assessed to confirm that the dataset conformed to normal distribution assumptions. Subsequently, the reliability and validity of the measurement instruments were evaluated, with Cronbach’s alpha coefficients used to establish internal consistency and confirmatory factor analysis employed to validate the measurement model. Descriptive statistics further illuminated the central tendencies and variability of the data, offering a clearer view of key patterns. SPSS facilitated an in-depth exploration of the relationships between fundamental psychological needs, resilience, flow, and engagement in the IDLE context. Finally, SEM was used to examine the hypothesized pathways, shedding light on potential mediating effects and the dynamics between constructs.

The qualitative analysis, conducted with NVivo 12.0, followed a structured process to complement the quantitative findings. The analysis began with a detailed review and translation of participants’ responses to ensure clarity, accuracy, and contextual understanding. Once the data were prepared, the coding phase involved identifying key units, including words, phrases, and sentences, which were systematically categorized into thematic nodes corresponding to relevant factors. This coding process enabled the emergence of nuanced themes that captured participants’ experiences and perspectives. In the next phase, a detailed thematic synthesis was conducted by examining the frequency and interconnectedness of the nodes. This analysis provided insights into learners’ engagement with IDLE, highlighting recurring patterns and novel perspectives that aligned with the research questions. Moreover, NVivo’s visualization tools facilitated the identification of relationships between themes, enriching the overall analysis. By integrating quantitative and qualitative findings, this dual-method approach offered a comprehensive understanding of the factors influencing EFL learners’ engagement in IDLE.

## 4. Results

### 4.1. Quantitative Analysis

#### 4.1.1. Descriptive Statistics

Table 1 provides an overview of several constructs related to student engagement, resilience, flow, and basic psychological needs. Emotional, cognitive, and behavioral engagement items show mean scores ranging from 2.94 to 3.81, suggesting moderate to high levels of engagement with IDLE among participants. The score of resilience items all exceeding 3.2, suggesting that learners demonstrate robust adaptability, persistence, and emotional regulation when encountering challenges or setbacks during informal digital language learning activities. The flow items with an average score above 3.2 suggests that learners were more likely to perceive their activities as enjoyable, fulfilling, and intrinsically rewarding. Lastly, mean scores for basic psychological needs exceed 3.3 among Chinese English learners, which indicates a favorable situation regarding their engagement with the IDLE.

#### 4.1.2. Reliability, Validity, and the Measurement Model

This section systematically presents the results related to reliability, multivariate normality, convergent validity, discriminant validity, and the overall measurement model. The reliability of the scales used in the study was assessed by calculating Cronbach’s α values for nine variables, including emotional engagement, cognitive engagement, behavioral engagement, resilience, perceived autonomy, perceived competence, and perceived relatedness. As displayed in Table 1, Cronbach’s α values ranged from 0.77 to 0.92, which exceeded the widely accepted threshold of 0.70 (Kline, 2023), indicating that the scales demonstrated sufficient internal consistency and reliability for the variables measured. This robust internal consistency supports the credibility of the data collected, making it suitable for further statistical analyses.

The study also evaluated multivariate normality and sampling adequacy to determine the suitability of the dataset for factor analysis. The Kaiser–Meyer–Olkin (KMO) measure and Bartlett’s test of sphericity were used to assess these criteria. The KMO value, which measures sampling adequacy, was 0.77, indicating that the dataset was appropriate for factor analysis as it exceeded the recommended minimum threshold of 0.6 (Tabachnick & Fidell, 2019). Additionally, Bartlett’s test of sphericity yielded a significant result (*p* < 0.001), further affirming the adequacy of the data for such analyses. These results suggest that the correlations among the variables were sufficiently high to justify factor analysis. Furthermore, univariate skewness and kurtosis were calculated for all scale items to assess normality, with results indicating that the absolute values of skewness were below 2 and those of kurtosis were below 10, as shown in Table 2. These findings align with the recommendations of Collier (2020) for establishing multivariate normality. Thus, the results provide a strong foundation for performing further analyses, ensuring that the data meet the necessary assumptions for validity testing.

To evaluate the validity of the constructs, confirmatory factor analysis (CFA) was conducted, focusing on convergent and discriminant validity. Convergent validity was assessed using composite reliability (CR) and average variance extracted (AVE) for each factor. The CR values exceeded the recommended threshold of 0.7, while AVE values surpassed the benchmark of 0.5, as shown in Table 3. These results indicate that the constructs were well-explained by their respective indicators and shared sufficient variance with them (Kline, 2023). Establishing convergent validity ensures that the measurement items effectively represent the underlying constructs they were designed to measure. Discriminant validity was examined by comparing the square roots of AVE values with inter-factor correlation coefficients. The square roots of AVE values were greater than the highest squared inter-construct correlations, which ranged from 0.07 to 0.52 (see Table 2). This finding confirms that the constructs were distinct from one another, thereby satisfying the requirements for discriminant validity. Together, these validity tests validate the measurement model, demonstrating its ability to accurately represent the hypothesized relationships among constructs. This validation is crucial for the credibility of subsequent structural equation modeling and hypothesis testing.

To evaluate the validity of the constructs, confirmatory factor analysis (CFA) was conducted, focusing on convergent and discriminant validity. Convergent validity was assessed using composite reliability (CR) and average variance extracted (AVE) for each factor. The CR values exceeded the recommended threshold of 0.7, while AVE values surpassed the benchmark of 0.5, as shown in Table 2. These results indicate that the constructs were well explained by their respective indicators and shared sufficient variance with them (Kline, 2023). Establishing convergent validity ensures that the measurement items effectively represent the underlying constructs they were designed to measure. Discriminant validity was examined by comparing the square roots of AVE values with inter-factor correlation coefficients. The square roots of AVE values were greater than the highest squared inter-construct correlations, which ranged from 0.07 to 0.52 (see Table 2). This result confirms that the constructs were distinct from one another, thereby satisfying the requirements for discriminant validity. Together, these validity tests validate the measurement model, demonstrating its ability to accurately represent the hypothesized relationships among constructs. 

To further evaluate the construct validity of the proposed measurement model, an analysis was conducted using AMOS 26.0. A robust measurement model is essential for ensuring that the observed variables effectively represent the underlying latent constructs. To assess the model fit, seven widely recognized goodness-of-fit indices were calculated, including the comparative fit index (CFI), normed fit index (NFI), incremental fit index (IFI), root mean square error of approximation (RMSEA), Tucker–Lewis index (TLI), standardized root mean squared residual (SRMR), and Parsimony normed fit index (PNFI). These indices collectively offer a comprehensive evaluation of how well the model aligns with the observed data. As shown in Table 3, the computed values for each fit index indicated that the model provided an excellent fit to the data, with all indices exceeding or falling within the recommended thresholds specified in the literature (Collier, 2020; Kline, 2023).

#### 4.1.3. The Structural Model and Hypotheses Testing

**Figure 2:**
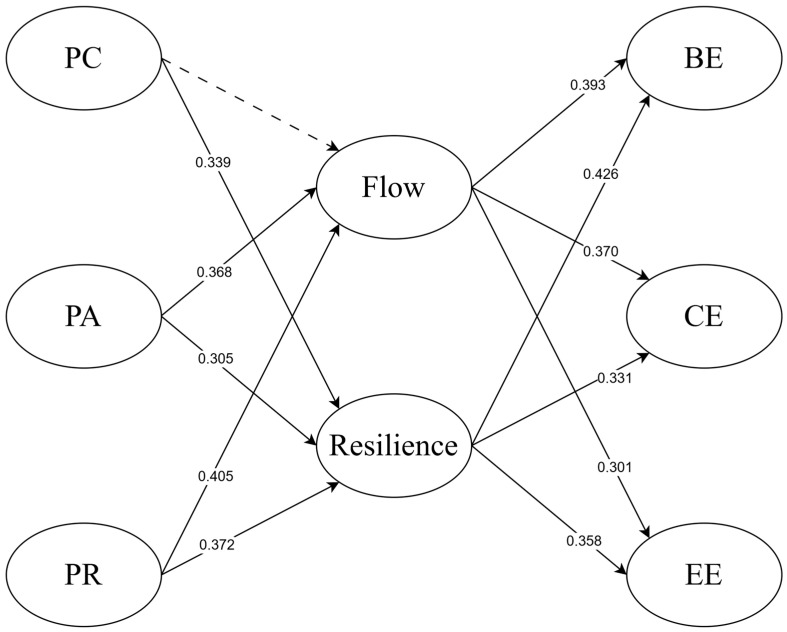
The Final Structural Model. A dotted arrow indicates that the path is not significant.

Building on the established measurement model, we tested the full structural model, which demonstrated a good fit based on the goodness-of-fit statistics as shown in Figure 2. Figure 2 illustrates the structural relationships between perceived competence, perceived autonomy, and perceived relatedness as predictors, and flow and resilience as mediators, leading to three forms of engagement: behavioral engagement, cognitive engagement, and emotional engagement. The path coefficients presented in Figure 2 indicate the strength and significance of these relationships, showing the direct and indirect effects of the constructs. Summarizing the major results of the path analysis, Table 4 details the results of the 12 hypotheses. Table 4 provides a summary of the standardized path coefficients, significance levels, and hypothesis testing results, highlighting which relationships were supported and which were rejected.

Table 5 presents the results of the mediation analysis, examining the indirect effects of perceived competence, perceived autonomy, and perceived relatedness on behavioral engagement, cognitive engagement, and emotional engagement through the mediators—flow and resilience. The analysis reports the 95% confidence intervals, *p*-values, and indirect effect estimates for each pathway. Indirect effects are deemed significant if the confidence intervals do not include zero and the *p*-values are below 0.05. The results indicate that perceived autonomy and perceived relatedness exhibit significant indirect effects on all three forms of engagement (behavioral, cognitive, and emotional) via flow and resilience, while perceived competence does not demonstrate significant indirect effects through flow, as evidenced by confidence intervals crossing zero.

### 4.2. Qualitative Analysis

#### 4.2.1. Understanding the Mediating Role of Resilience in IDLE

In the context of IDLE, resilience acts as a vital mediator, encompassing five key themes: adaptability enhancer, persistence promoter, stress buffer, self-efficacy builder, and emotional regulation facilitator. These themes illustrate the comprehensive role of resilience in supporting learners as they navigate the challenges of the informal digital learning environments. The subsequent analysis elaborates on these themes, demonstrating how resilience mediates the relationship between basic psychological needs and learner engagement in IDLE. The details are as follows.

Firstly, resilience helps learners adjust to different learning scenarios, tools, and potential disruptions inherent in IDLE, promoting an adaptive mindset that is crucial for navigating the self-regulated learning paths. This adaptability ensures that learners can embrace the evolving nature of digital learning platforms and the diversity of educational content available. The following excerpts reveal resilience as a key enabler of learner adaptability in IDLE:

P1: I’ve learned to switch between different platforms depending on what I need to focus on. Flexibility is key.

P20: Sometimes the internet is down, or a site crashes, but I’ve gotten good at finding new ways to practice my English.

Secondly, resilience instills a sense of determination within learners, galvanizing them to persevere through challenges encountered in IDLE. It is this resilience-driven tenacity that underpins continuous engagement and dedication to learning, allowing learners to view obstacles as stepping stones along their educational journey. The following excerpts highlight resilience driving learner persistence despite digital learning challenges:

P3: I hit a wall with grammar, but I kept at it every day, and it’s starting to make sense now.

P18: Every challenge in learning English online is just a hurdle, not a stop sign. I jump over it and keep running.

Thirdly, resilience acted as a protective shield to mitigate the stress associated with the autonomy of IDLE, particularly when learners face the daunting task of self-management and the potential for feeling adrift in a sea of digital information. By buffering the emotional toll of such stressors, resilience enables learners to approach learning with a more centered disposition. The following excerpts illustrate resilience buffering stress in IDLE contexts:

P2: If I start to feel anxious about all the new words, I remind myself that it’s a process, and I just need to take it one step at a time.

P19: Learning English used to stress me out, but now I set small goals, and it’s less intimidating.

Fourthly, cultivating resilience fortifies learners’ confidence in their own abilities, thereby enhancing their self-efficacy. This reinforced self-belief is pivotal, as it propels learners towards embracing more ambitious learning targets, fostering a proactive and self-assured educational experience. The following excerpts reflect resilience boosting learners’ confidence in mastering English digitally:

P5: Mastering a difficult topic really boosts my confidence. It shows me what I’m capable of.

P16: Seeing my progress in the app’s tracking system gives me the confidence to keep pushing forward.

Lastly, resilience serves as a regulatory beacon, guiding learners through the emotional turbulence that can accompany language learning. This guidance is crucial for sustaining a learner’s journey through the highs and lows, enabling a balanced and positive approach to language acquisition. The following excerpts show resilience aiding learners in regulating emotions during language acquisition:

P4: When I get frustrated, I remember why I started learning English, and it helps me push through the negative feelings.

P14: I used to get really upset when I couldn’t understand a listening exercise, but now I just give it time and try again.

#### 4.2.2. Understanding the Mediating Role of Flow in IDLE

In the context of IDLE, flow emerges as a critical mediator that enhances engagement through four overarching themes: intrinsic motivation driver, positive cycle creator, external pressure mitigator, and efficiency enhancer. These themes encapsulate the multifaceted role of flow in informal digital language learning, highlighting its contribution to fostering sustained engagement and optimizing learning experiences. The subsequent analysis delves into each theme, illustrating how flow mediates the relationship between basic psychological needs and learner engagement in IDLE. The detailed analysis is as follows:

Firstly, the experience of flow acts as a key driver of intrinsic motivation in IDLE, leading students to engage in learning for the pleasure and satisfaction derived from the learning itself. This high motivational state induced by flow not only encourages students to delve deeper into English learning content but also strengthens their willingness to continue learning in informal settings. The excerpts below reveal how flow serves as an intrinsic motivation driver:

P1: Every time I figure out a new word by myself, it’s a win. It makes me want to dive deeper, not just for grades but for the thrill of it.

P16: Learning English this way just clicks with me. It’s satisfying to see my progress, and it pushes me to set higher goals for myself.

Secondly, flow helps students establish a positive learning cycle by offering positive learning experiences. When students experience flow in IDLE, they are more likely to seek similar experiences, thereby increasing their engagement in IDLE while satisfying their basic psychological needs. These excerpts highlight the role of flow as a positive cycle creator:

P3: After completing a challenging puzzle in English, I felt so accomplished, I immediately wanted to tackle another one.

P11: The feedback I get on my progress is always so positive; it makes me want to keep improving.

Thirdly, flow in IDLE environments aids students in focusing on the learning process, thereby minimizing the impact of external distractions. This complete immersion helps students overcome challenges in learning, enhancing their ability to continue learning in the face of difficulties, and thus promoting deeper levels of engagement. The following excerpts demonstrate that flow plays the role of external pressure mitigator: 

P4: Whenever I feel overwhelmed, I switch to learning English online. It’s like a breath of fresh air, away from the stress of college life.

P12: It’s my escape from the noise. I can be anywhere, plug in my headphones, and I’m in my own world, learning and growing.

Lastly, the state of flow increases students’ engagement in IDLE and enhances the value of this engagement by improving learning efficiency and outcomes. When students experience flow, they can process learning materials more effectively and achieve learning goals, encouraging further exploration and utilization of informal learning resources. These excerpts illuminate that flow plays the role of efficiency enhancer:

P2: I can learn twice as much in half the time with these apps. They make learning efficient and effective.

P19: These learning platforms have a way of breaking down complex concepts into digestible pieces, making it easier to grasp and remember.

## 5. Discussion

### 5.1. Resilience in IDLE: Buffering Stress and Promoting Engagement

Regarding the mediating role of resilience in research question one, our discussions are as follows. Perceived relatedness, perceived autonomy, and perceived competence significantly predict resilience in IDLE. This finding is in line with existing research indicating that when students perceive a strong sense of connection with others (relatedness), feel in control of their learning (autonomy), and believe in their capabilities (competence), they are better equipped to withstand challenges (Liu & Huang, 2021; Neufeld & Malin, 2019). Our study extends this field by empirically validating these constructs as predictors of resilience among EFL learners in an informal digital setting. Furthermore, resilience has been recognized as a crucial predictor of behavioral, cognitive, and emotional engagement. This relationship resonates with the findings from studies by Liu et al. (2022, 2023c) and Ritonga et al. (2023), which illustrate resilience’s pivotal role in shaping various facets of engagement. Furthermore, resilience serves as a stress buffer, mitigating the impact of anxiety and frustration often associated with mastering a new language. As a self-efficacy builder, resilience strengthens learners’ belief in their capabilities, which is crucial for the sustained effort and engagement required for language proficiency. Lastly, resilience functions as an emotional regulation facilitator, equipping learners with the tools to manage emotional responses effectively. Together, these roles underscore the essential function of resilience as a mediator, not only reinforcing the link between basic psychological needs and engagement but also highlighting its crucial contribution to the robustness and adaptability of the language learning process.

In aligning our qualitative findings with the existing literature, the multifaceted role of resilience as a mediator between basic psychological needs and engagement is further validated. The conceptualization of resilience as an adaptability enhancer echoes the assertions of Cena et al. (2023), who highlight resilience’s role in facilitating learners’ adaptability to changing educational contexts. Similarly, our identification of resilience as a persistence promoter aligns with the work of Kim et al. (2019), who emphasize the critical role of resilience in sustaining student motivation and perseverance in academic pursuits. The stress-buffering capacity of resilience, as observed in our study, resonates with the findings of Shen (2022), illustrating how resilience ameliorates the detrimental effects of stress and anxiety on learning. In terms of enhancing self-efficacy, our results parallel Li’s (2022) discussion on the importance of resilience in building self-efficacy, further corroborating the significance of resilience in fostering an enduring belief in one’s abilities. Moreover, our analysis on resilience as an emotional regulation facilitator finds support in Kim et al.’s (2018) research, which outlines the importance of emotional regulation strategies in managing learning-related emotions. This comparison with the existing literature not only reinforces the validity of our findings but also highlights the integral role of resilience in bridging basic psychological needs and engagement, enriching the discourse on effective language learning strategies.

The findings highlight three key pedagogical implications for fostering resilience in informal digital learning environments. First, creating opportunities for learners to engage with others, such as through discussion forums, collaborative online tasks, or community groups, can strengthen perceived relatedness, autonomy, and competence, which are critical for building resilience. Second, resilience should be nurtured as it helps mitigate stress and anxiety, often encountered in informal learning contexts. Learners can be encouraged to adopt self-care strategies, such as reflective journaling or time management practices, to enhance their emotional well-being. Third, resilience supports self-efficacy and emotional regulation, which are essential for maintaining engagement in informal settings. Digital platforms can integrate features like progress tracking, motivational messages, or peer support to help learners stay confident and manage their emotions effectively. These strategies align with the self-directed nature of informal learning, empowering learners to adapt and thrive in unstructured digital environments.

### 5.2. Flow in IDLE: Fostering Intrinsic Motivation and Enhancing Engagement

In terms of the mediating role of flow in research question two, our discussions are as follows. Our findings indicate that both perceived relatedness and perceived autonomy serve as predictors of flow, aligning with prior research (Stormoen et al., 2016; Valenzuela et al., 2018). This consistency underscores the significance of social connections and self-directed learning in facilitating a state of deep immersion and engagement in language learning activities. However, contrary to the observations by He et al. (2023) in the context of formal entrepreneurship courses, perceived competence did not emerge as a predictor of flow in our study, which focuses on IDLE. This discrepancy suggests that the role of perceived competence in predicting flow may vary significantly between formal learning environments and informal learning settings. In IDLE, the absence of perceived competence as a predictor of flow can be attributed to the self-paced and exploratory nature of these learning environments. Without the pressure of formal assessments and structured curricula, learners are less likely to equate their competence with their ability to achieve flow. This context shifts the focus from performance and proficiency to personal interest and connection, diluting the impact of perceived competence on the flow experience. Flow has also been identified as a predictor of behavioral, cognitive, and emotional engagement, a finding that aligns with previous research (Hamari et al., 2016; Santosa, 2015). This relationship underscores the significant role of flow in enhancing the depth and quality of engagement with language learning.

The mediating role of flow, as revealed through our qualitative analysis, aligns with and extends existing literature on the subject. The identification of flow as an intrinsic motivation driver corroborates findings by Li et al. (2021) who emphasized flow’s capability to enhance motivation by engaging learners in activities that are optimally challenging and rewarding. Our analysis, revealing flow as a positive cycle creator, expands on the findings of Santosa (2015), who suggest that flow experiences can lead to a virtuous cycle of engagement and performance enhancement. Contrasting with our findings, the role of flow as an external pressure mitigator offers a nuanced perspective compared to the broader discussions by Csikszentmihalyi (1997), who specifically highlights how flow in language learning contexts alleviates the pressures and anxieties unique to acquiring new languages. This specificity adds depth to the understanding of flow’s buffering effects against external stressors in educational settings. Lastly, the efficiency enhancement attributed to flow in our study complements the work of Wu et al. (2022) who discussed flow’s impact on performance and learning efficiency. We extend this narrative by detailing how efficiency gains through flow are manifested in the informal context, underscoring the significance of flow for effective language acquisition.

Based on the findings, three key pedagogical implications for fostering flow in informal digital learning environments emerge. First, promoting social connections and creating opportunities for self-directed learning are critical for facilitating flow. Educators and platform designers can encourage collaborative activities or interactive features, such as discussion forums or peer feedback, to enhance perceived relatedness and autonomy. Second, flow should be nurtured as an intrinsic motivator to deepen engagement. Learners can be guided to select activities that are optimally challenging and personally interesting, helping them to immerse themselves in the learning process. Third, strategies to reduce external pressures and anxieties should be integrated into informal digital platforms. This may include offering flexible learning paths and tools that help learners track progress without feeling overwhelmed. These approaches emphasize the self-paced, exploratory nature of informal learning, empowering learners to achieve meaningful and sustained engagement.

## 6. Conclusions

In conclusion, this study showed that resilience fully mediates the relationship between basic psychological needs and engagement in language learning. Resilience emerges as a multifaceted enhancer of adaptability, promoting persistence, buffering stress, building self-efficacy, and facilitating emotional regulation. These roles underscore resilience’s comprehensive impact on sustaining engagement by enabling learners to navigate the challenges and dynamics of informal digital learning environments effectively. Conversely, flow partially mediates this relationship, with perceived competence not predicting flow in this context. Flow contributes significantly to the language learning process by driving intrinsic motivation, creating a self-reinforcing cycle of engagement, mitigating external pressures that may impede learning, and enhancing the efficiency of the learning experience. Together, these mediating roles of resilience and flow illuminate the complex interplay of basic psychological factors that influence engagement in IDLE among Chinese university EFL students. These findings provide valuable insights for educators and policymakers aiming to design effective strategies to enhance learner engagement in informal digital environments.

This study is not without limitations. Firstly, the reliance on online questionnaires as the sole method for quantitative data collection may have limited the ability to capture the full range of participants’ experiences and perspectives, potentially reducing the comprehensiveness of the data. Secondly, the research does not incorporate methods such as observations or learner diaries, which could provide richer and more nuanced insights into learners’ experiences with resilience and flow. Future research should explore the integration of diverse data collection techniques to address these limitations. Additionally, it would be valuable to investigate the role of contextual and cultural factors in shaping the dynamics of resilience and flow in IDLE. Longitudinal studies could provide insights into the long-term trajectories of these constructs, uncovering key developmental phases or external influences that affect learner engagement over time. These advancements would contribute to a deeper understanding of how resilience and flow evolve in IDLE contexts, paving the way for more targeted and effective educational interventions.

## Figures and Tables

**Table 1:** Descriptive statistics.

Constructs	Items	M	SD	Kurtosis	Skewness	α
Emotional Engagement	EE1	3.43	0.97	−0.37	−0.18	
	EE2	3.28	1.04	−0.44	−0.21	0.84
	EE3	3.74	0.94	−0.16	−0.48	
Cognitive Engagement	CE1	2.94	0.92	−0.30	0.01	
	CE2	3.50	1.02	−0.44	−0.29	0.83
	CE3	3.17	1.04	−0.36	−0.22	
Behavioral Engagement	BE1	3.06	0.96	−0.40	0.08	
	BE2	3.36	0.97	−0.30	−0.23	0.86
	BE3	3.81	0.84	−0.27	−0.31	
Resilience	R1	3.43	1.02	−0.46	−0.20	
	R2	3.56	1.03	−0.66	−0.25	
	R3	3.60	0.92	−0.31	−0.30	0.92
	R4	3.49	0.99	−0.50	−0.20	
	R5	3.27	0.97	−0.28	−0.20	
	R6	3.23	1.01	−0.50	−0.02	
Flow	F1	3.27	1.05	−0.61	−0.08	
	F2	3.46	0.96	−0.55	−0.16	
	F3	3.84	0.88	−0.21	−0.44	0.88
	F4	3.60	1.01	−0.45	−0.34	
	F5	3.61	0.91	−0.22	−0.26	
Perceived Relatedness	PR1	3.60	0.91	−0.34	−0.23	
	PR2	3.36	1.00	−0.37	−0.18	0.77
	PR3	3.53	0.94	−0.38	−0.28	
Perceived Autonomy	PA1	3.33	1.01	−0.58	−0.08	
	PA2	3.68	0.94	−0.47	−0.30	0.78
	PA3	3.57	0.98	−0.67	−0.14	
Perceived Competence	PC1	3.52	1.02	−0.36	−0.32	
	PC2	3.54	1.06	−0.52	−0.33	0.80
	PC3	3.71	0.85	−0.16	−0.26	

**Table 2:** Convergent validity and discriminant validity.

AVE	CR	Constructs	EE	CE	BE	R	F	PR	PA	PC
0.64	0.84	EE	**0.80**							
0.62	0.83	CE	0.28	**0.78**						
0.67	0.86	BE	0.28	0.38	**0.82**					
0.66	0.92	R	0.43	0.42	0.52	**0.81**				
0.60	0.88	F	0.39	0.45	0.49	0.23	**0.78**			
0.52	0.77	PR	0.24	0.24	0.35	0.35	0.40	**0.72**		
0.54	0.78	PA	0.23	0.21	0.25	0.28	0.37	−0.01	**0.73**	
0.59	0.81	PC	0.07	0.07	0.09	0.31	−0.03	−0.05	−0.06	**0.77**

Note: (1) The square root of AVE is demonstrated along the diagonal line in the bold. (2) EE = emotional engagement; CE = cognitive engagement; BE = behavioral engagement; R = resilience; F = flow; PR = perceived relatedness; PA = perceived autonomy; PC = perceived competence. (3) AVE = average variance extracted; CR = composite reliability.

**Table 3:** Goodness-of-fit indices of the measurement models.

	X^2^/df	CFI	NFI	IFI	RMSEA	TLI	SRMR	PNFI
Our Model	1.15	0.99	0.95	0.99	0.02	0.99	0.03	0.94
RV	<5	>0.90	>0.90	>0.90	<0.10	>0.90	<0.05	>0.5

Note: RV = recommended values; CFI = comparative fit index; NFI = normed fit index; IFI = incremental fit index; RMSEA = root mean square error of approximation; TLI = Tucker–Lewis index; SRMR = standardized root mean squared residual; PNFI = Parsimony normed fit index.

**Table 4:** Hypotheses test results.

Hypotheses	β	*P*	t	S.E.	Results
H1: PC→F	0.008	0.863	0.172	0.055	Rejected
H2: PC→R	0.339	***	6.982	0.058	Accepted
H3: PA→F	0.368	***	6.992	0.06	Accepted
H4: PA→R	0.305	***	6.168	0.057	Accepted
H5: PR→F	0.405	***	7.548	0.062	Accepted
H6: PR→R	0.372	***	7.305	0.06	Accepted
H7: F→BE	0.393	***	8.483	0.047	Accepted
H8: F→CE	0.370	***	7.365	0.046	Accepted
H9: F→EE	0.301	***	6.131	0.047	Accepted
H10: R→BE	0.426	***	9.362	0.046	Accepted
H11: R→CE	0.331	***	6.854	0.043	Accepted
H12: R→EE	0.358	***	7.342	0.046	Accepted

Note: (1) *p* < 0.001 = ***; (2) EE = emotional engagement; CE = cognitive engagement; BE = behavioral engagement; R = resilience; F = flow; PR = perceived relatedness; PA = perceived autonomy; PC = perceived competence.

**Table 5:** The mediation analysis.

Hypotheses	95% Confidence Interval		p (Two-Tailed Significance)	Indirect Effect	Results
	Lower Bound	Upper Bound			
PC→F→BE	−0.28	0.045	0.836	0.004	rejected
PC→F→CE	−0.26	0.037	0.876	0.004	rejected
PC→F→EE	−0.22	0.032	0.836	0.003	rejected
PA→F→BE	0.122	0.231	0.009	0.144	accepted
PA→F→CE	0.100	0.191	0.012	0.137	accepted
PA→F→EE	0.077	0.163	0.013	0.111	accepted
PR→F→BE	0.135	0.242	0.014	0.156	accepted
PR→F→CE	0.118	0.220	0.006	0.148	accepted
PR→F→EE	0.096	0.190	0.006	0.120	accepted
PC→R→BE	0.115	0.223	0.018	0.146	accepted
PC→R→CE	0.085	0.175	0.005	0.112	accepted
PC→R→EE	0.099	0.190	0.011	0.122	accepted
PA→R→BE	0.114	0.199	0.007	0.129	accepted
PA→R→CE	0.072	0.150	0.007	0.099	accepted
PA→R→EE	0.087	0.162	0.010	0.108	accepted
PR→R→BE	0.141	0.247	0.007	0.159	accepted
PR→R→CE	0.093	0.177	0.005	0.122	accepted
PR→R→EE	0.117	0.210	0.004	0.133	accepted

Note: EE = emotional engagement; CE = cognitive engagement; BE = behavioral engagement; R = resilience; F = flow; PR = perceived relatedness; PA = perceived autonomy; PC = perceived competence.

## Data Availability

The data presented in this study can be made available upon reasonable request from the corresponding author.
